# 10-km passive drone detection using broadband quantum compressed sensing imaging

**DOI:** 10.1038/s41377-025-01878-y

**Published:** 2025-07-14

**Authors:** Shuxiao Wu, Jianyong Hu, Jiaqing Ge, Yanshan Fan, Zhexin Li, Liu Yang, Kai Song, Jiazhao Tian, Zhixing Qiao, Guosheng Feng, Xilong Liang, Changgang Yang, Ruiyun Chen, Chengbing Qin, Guofeng Zhang, Liantuan Xiao, Suotang Jia

**Affiliations:** 1 State Key Laboratory of Quantum Optics Technologies and Devices, Institute of Laser Spectroscopy, Shanxi University, Taiyuan, 030006 China; 2 Collaborative Innovation Center of Extreme Optics, Shanxi University, Taiyuan, 030006 China; 3 College of Physics, Taiyuan University of Technology, Taiyuan, 030600 China; 4 College of Medical Imaging, Shanxi Medical University, Taiyuan, 030001 China; 5 Department of Materials and Chemical Engineering, Taiyuan University, Taiyuan, China

**Keywords:** Imaging and sensing, Quantum optics

## Abstract

Remote passive drone detection in the presence of strong background noise is challenging, since they are point objects and cannot be recognized by their contour detection. In this study, we introduce a new passive single-photon dynamic imaging method using quantum compressed sensing. This method utilizes the inherent randomness of photon radiation and detection to construct a compressive imaging system. It captures the broadband dynamic features of the point object through sparse photon detection, achieving a detectable bandwidth up to 2.05 GHz, which is significantly higher than current photon-counting imaging techniques. The method also shows excellent noise resistance, achieving high-quality imaging with a signal-to-background ratio of 1/332. This technique significantly enhances the use of single-photon imaging in real-world applications.

## Abstract (web summary)

The dynamic characteristics of drone are detected at the single-photon level.

## Graphical abstract

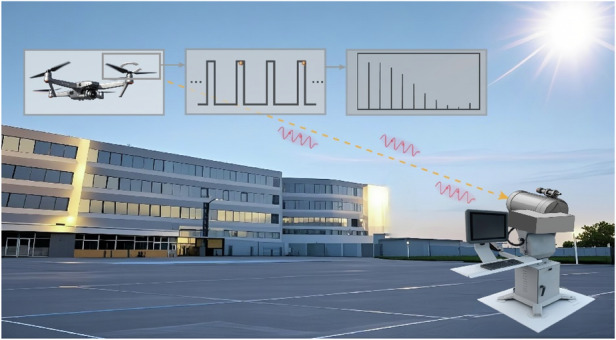

## Introduction

The use of drones has significantly boosted low-altitude economic activities, making drone monitoring a necessity. However, detecting drones remotely is challenging due to weak light signals, strong background noise, and small scattering cross sections. Single-photon imaging (SPI) is useful for detecting weak objects with high sensitivity, valuable in remote sensing, disaster relief, and astronomy^1–7^. However, traditional passive SPI methods, which rely on spatial photon distribution, struggle with noise and are slow at capturing dynamic targets^8–11^.

Further, applications in extreme environments need better imaging systems with enhanced dynamic imaging and noise resistance. Current dynamic SPI techniques, which use time-correlated single-photon counting^12–19^, require active illumination and synchronization, limiting their use in passive systems^20–22^. These systems also fail to capture the high-frequency dynamics of non-cooperative objects due to long photon-counting integration times. Consequently, dynamic characteristics regarding remote sensing objects becomes unattainable for accurate object recognition. This issue is particularly exacerbated when detecting distant objects that occupy only a limited number of pixels in an image.

This study introduces a broadband quantum compressed sensing (QCS) imaging method that utilizes the quantum randomness of photon radiation and detection to construct a compressed sensing system, enabling the capture of dynamic target features through sparse photon detections. The background noise appears as white noise in the frequency domain, which gives it a constant power spectral density. Therefore, this method demonstrates excellent resistance to noise by extracting dynamic features in the frequency domain of the objects. Experiments showed the QCS imaging system, using a SPAD array^23^, could detect dynamic frequency up to 2.05 GHz with a data compression ratio of 1.95 × 10^−6^ and an impressive imaging signal-to-background ratio (SBR) of 1/332.

### Quantum compressed sensing imaging

To obtain dynamic information about high-speed moving or rotating objects, conventional imaging techniques typically attempt to increase the image frame rate. However, the photon-counting-based SPI method needs a long integration time to accumulate sufficient photon counts to ensure an acceptable signal-to-noise ratio (SNR)^8,10,24^. Even with compressed sensing (CS) imaging technology, such as the combination of a single-pixel detector and a digital micromirror device (DMD), which has improved the imaging frame rate to tens of Hz, capturing higher frequency dynamic information remains limited by both the switching rate of the DMD and the photon count rate^25–27^. Therefore, simply increasing the frame rate for capturing dynamic object information in SPI is not a viable solution.

This study proposes a passive QCS imaging method, enabling the direct extraction of broadband dynamic information of the objects. Here, QCS can be defined as a signal processing technique for efficiently acquiring and reconstructing signals from considerably fewer samples than required by the Nyquist-Shannon theorem by constructing a compressive measurement system using quantum resources, such as quantum coherence, quantum entanglement, etc^21,28–30^. Reference^21^ achieved GHz-level frequency-domain imaging using a near-infrared up-conversion imaging system. It directly extracts the flicker frequency of objects via discrete Fourier transform and employs the spectral peaks for imaging, thereby effectively suppressing background noise. In this study, a comprehensive QCS theoretical model is established, enabling the waveform and image reconstruction of moving objects. The implementation of QCS can be divided into four steps: initial quantum state preparation, quantum state manipulation, quantum state detection and signal reconstruction. The mathematical model of QCS can be described as follows:

1 $$y=\hat{A}|{{\psi }}^{x}\rangle$$

where |*ψ*〉 represents the initial quantum state; *x* denotes the signal to be measured, which is sparse in some transform domain; *Â* denotes the measurement operator, and *y* represents the measurement result. The quantum state |*ψ*^*x*^〉 corresponds to the process of manipulating quantum state |*ψ*〉 with signal *x*. Then, the measurement operator *Â* acts on the quantum state |*ψ*^*x*^〉, corresponding to the process of quantum state detection. Finally, signal reconstruction can be achieved by employing a CS reconstruction algorithm based on the measurement result *y* (see Supplementary Note 1 for more detailed QCS theory description).

**Fig. 1:**
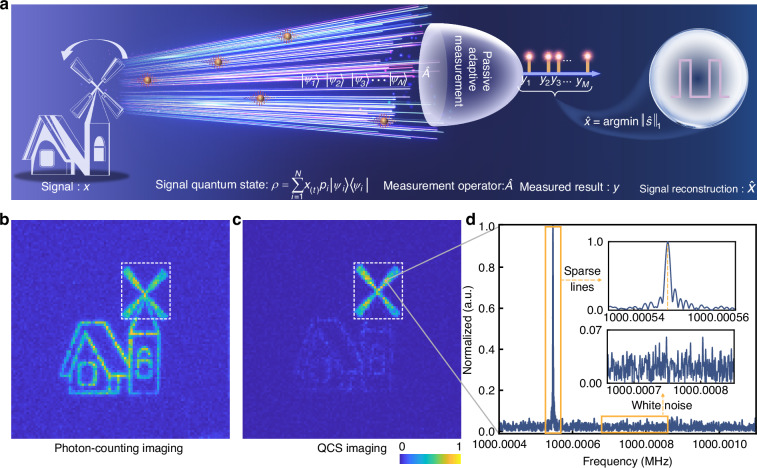
Principle illustration of the QCS imaging. **a** Principle diagram of the passive QCS imaging approach, see the Materials and methods for experimental details. The radiation signal *x* of the windmill can be considered as a modulated mixed state that corresponds to the initial quantum state preparation and manipulation in QCS. Our goal is to reconstruct the signal through sparse photon detection, where time-domain single-photon detection corresponds to the process of quantum states detection, and finally obtain the estimated signal through a signal reconstruction algorithm. **b** The photon-counting image of a simulated windmill house pattern. **c** QCS imaging result. **d** Dynamic characteristic spectrum of the signal. Each pixel records the photon arrival time independently, and the characteristic spectrum can be obtained by performing a discrete Fourier transform on the photon arrival time series *y* = [*y*_*1*_, *y*_*2*_, …, *y*_*m*_, …, *y*_*M*_]. Signal *x* can be reconstructed by applying a signal reconstruction algorithm, which will show in Fig. 4. Here, we demonstrate the feasibility of our method solely through a simulated pattern. The two illustrations depict sparse characteristic spectrum lines and a white noise base derived from background noise counts, dark counts, and shot noise of photon counting

Specifically, in this work, as the proposed approach is a passive imaging method, the object radiation field consists of ambient scattered light. Therefore, the initial quantum state is a mixed state. Considering that the object exhibits high-frequency rotational dynamics and its signal is sparse in the frequency domain (e.g., like the windmill shown in Fig. 1a), the signal can be represented by a time-varying pulse waveform *x*_(*t*)_. Consequently, the quantum state manipulated by this signal is transformed into a time-varying mixed state, and its density operator can be expressed as:

2 $$\rho =\mathop{\sum }\limits_{i=1}^{N}{x}_{(t)}{p}_{i}|{\psi }_{i}\rangle \langle {\psi }_{i}|$$

where *p*_*i*_ represents the fraction of the ensemble in each pure state |*ψ*_*i*_〉, *N* is the Nyquist sampling rate. In the quantum state detection step, a photon is assigned to a specific pure state upon detection. In this work, we assume that the dynamic information of the object is sparse in the frequency domain, i.e., the object only presents finite discrete frequencies. Based on this assumption, a QCS system is constructed by using the inherent randomness of sparse photon radiation and detection. The measured result *y* = [*y*_*1*_, *y*_*2*_, …, *y*_*m*_ …, *y*_*M*_] (*M* < < *N*) corresponds to a time series of photon collapses, and the signal can be recovered by using the reconstruction algorithm^21,28–31^. Finally, an estimation of signal *x* is obtained by solving the following non-positive definite equation through an inverse Fourier transform:

3 $$\hat{x}={\text{arg}}\,\min {\Vert {\hat{s}}\Vert }_{1}$$

where *ŝ* is the estimate of non-zero elements of sparse coefficients. See Supplementary Note 2 for more detailed description of signal reconstruction algorithm. Experiments was performed, as shown in Fig. 1a. The results of a traditional photon-counting imaging system and the proposed QCS imaging system were evaluated (Fig. 1b, c). The QCS system captures a dynamic frequency of 1.0000005467 GHz, deviating from the set value of 1.0 GHz by 546.7 Hz due to the disparate clocks employed by the object and detection system. QCS imaging behaving like a filter that isolates the desired dynamic feature while filtering out other frequency components. A specific pixel from Fig. 1c is selected for spectrum analysis, the dynamic characteristic spectrum of the object exhibits sparsity in frequency domain, as depicted in Fig. 1d.

In contrast to conventional single-pixel imaging techniques that employ CS to increase the frame rate and resolution, QCS imaging systems directly capture the dynamic information of the object by using the quantum randomness of object photon radiation and single-photon detection. In conventional CS systems, the sensing matrix *A* is a predefined random matrix. Thus, its measurement is nonadaptive, meaning that the sampling process remains constant regardless of the signal. However, in the QCS system, the measurement results are obtained by acting the measurement operator on the quantum state. The randomness of the measurement matrix is derived from the inherent randomness of the collapse of the quantum state |*ψ*^*x*^〉. Since the quantum state |*ψ*^*x*^〉 is directly associated with the measured signal *x*, the measurement matrix is jointly determined by the initial state |*ψ*〉, the signal *x* and the measurement operator *Â*. Here, we assume that signal *x* is non-cooperative. Therefore, the QCS is a kind of passive adaptive measurements, which are impossible to obtain with conventional CS systems. For a detailed theoretical analysis, please refer to Ref.^29^. Supplementary Note 1 describes the key differences between QCS and CS in detail.

## Results

The proposed QCS imaging technique demonstrates excellent performance in measuring bandwidth and noise resistance. We quantitatively characterize its performance and demonstrate its feasibility in real-world environments through field drone tests.

### Bandwidth of QCS imaging

**Fig. 2:**
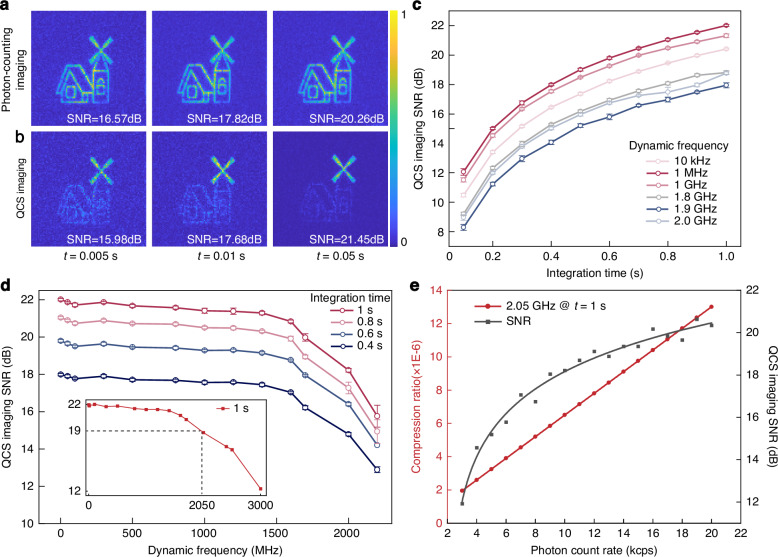
Performance characterization of the broadband QCS imaging. **a**, **b** The photon-counting imaging and QCS imaging results of the projected pattern. Three integration times were selected, and the SNR of the image was calculated for each case. **c** SNR of the QCS imaging system versus the integration time at a dynamic frequency ranging from 10 kHz to 3.0 GHz. **d** SNR of the QCS imaging system versus the dynamic frequency at integration times ranging from 0.4 s to 1 s. When the integration time is 1 s, the 3 dB bandwidth measured is 2.05 GHz. **e** Relationships between the data compression ratio and photon count rate, and between the QCS imaging SNR and photon count rate at a dynamic frequency bandwidth of 2.05 GHz and an integration time of 1 s. When the photon count rate is 3 kcps, the data compression ratio is 1.95 × 10^−6^

To demonstrate the ability of our method to capture dynamic features, an object with a broadband flicker was artificially constructed via laser projection, whose flicker frequency could be tuned from 10 kHz to 3.0 GHz. A comprehensive description of the experimental system is provided in the Materials and Methods. The projected pattern is a windmill house, with the windmill section exhibiting high-frequency flicker characteristics and the remaining area showing stationary characteristics. Here, the object is considered to be a non-cooperative entity. For comparison, we performed photon-counting imaging and QCS imaging. The photon-counting imaging method only records the photon count of each pixel within a specific integration time, whereas the QCS imaging enables dynamic feature extraction directly while suppressing non-dynamic regions in the scene, as depicted in Fig. 2a, b.

A quantitative analysis was conducted to investigate the factors that determine the imaging performance of QCS, including flicker frequency, integration time, and photon count rate. We analyzed their impact on SNR, bandwidth, and data compression ratio. As the integration time increases, the imaging SNR of the QCS imaging considerably increases, as depicted in Fig. 2c. The SNR is defined herein as^32^:

4 $$SNR=10\,{\log }_{10}\left(\frac{\lambda }{\sigma }\right)$$

where *λ* is the mean value of the signal and *σ* is the standard deviation of the noise. With the same photon count rate, increasing the integration time significantly improves the SNR. Conversely, when the integration time is held constant, increasing the flicker frequency leads to a decrease in the imaging SNR. Furthermore, the maximum detectable flicker frequency of the system was examined, as depicted in Fig. 2d. As the flicker frequency increased, the SNR remained consistent across a wide bandwidth range. In signal processing, the 3 dB bandwidth is a critical metric for evaluating the bandwidth performance of a measurement system. Here, the 3 dB bandwidth of the QCS imaging system is the frequency range corresponding to a 3 dB reduction of the SNR during an integration time of 1 s, i.e., 2.05 GHz, as shown in the subplot of Fig. 2d. Further increases in the detectable bandwidth are limited by the system time jitter^33^. The single-photon detector and time-to-digital converter (TDC) used in this study have time jitters of *t*_*r1*_ = 350 ps and *t*_*r2*_ = 200 ps, respectively. Consequently, the overall system time jitter is calculated as *t*_*r*_ = (*t*_*r1*_^2^ + *t*_*r2*_^2^)^1/2^ ≈ 403 ps. This results in a theoretical maximum effective detection bandwidth of 1/*t*_*r*_ ≈ 2.48 GHz. However, in practical experiments, the effective measurement bandwidth is diminished due to frequency distortions and scattering noise. However, the frame rate of the most advanced SPAD arrays can reach hundreds of kHz. However, limited by the integration time, storage depth and pixel scale, it is not mature in practical applications. The measurable bandwidth has been increased by six orders of magnitude when our QCS imaging system is compared to the advanced SPI technique^34–37^.

Data compression ratio is an important index to measure compressive sensing system, which is defined as the ratio of the compressed sampled data volume to the original sampled data volume, where the original sampled data refers to the Nyquist sampling. The data compression ratio of this work could be expressed as:

5 $$CR=\frac{({C}_{s}+{C}_{d})\times {d}_{a}\times t}{2\times B\times {d}_{b}\times t}$$

where *C*_*s*_ and *C*_*d*_ are the photon count rate of the signal and dark count, respectively; and *d*_*a*_ = 32 *bit* and *d*_*b*_ = 12 *bit* are the storage depths of the QCS imaging and traditional sampling systems, respectively; *B* is the signal bandwidth, *t* is the integration time. The relationship between the data compression ratio and the SNR of the QCS imaging system as a function of the photon count rate is illustrated in Fig. 2e. We varied the photon count rate of a single pixel from 3–20 kcps (counts per second), and the data compression ratio decreases as the photon count decreases. Since the bandwidth of QCS imaging is 2.05 GHz and its Nyquist sampling rate is 4.10 GHz, when the photon counting rate is 3.0 kcps, the data compression rate is 1.95 × 10^−6^ according to Eq. (5). The QCS imaging offers a novel approach to acquire broadband information by leveraging a limited number of photon detections, thereby overcoming the longstanding limitations of photon-counting imaging in dynamic object imaging. Its impressive technical indicators, such as achieving imaging bandwidth exceeding the GHz level and data compression ratio at the 10^−6^ level, signify a remarkable advancement in the field of imaging.

### Noise resistance of QCS imaging

**Fig. 3:**
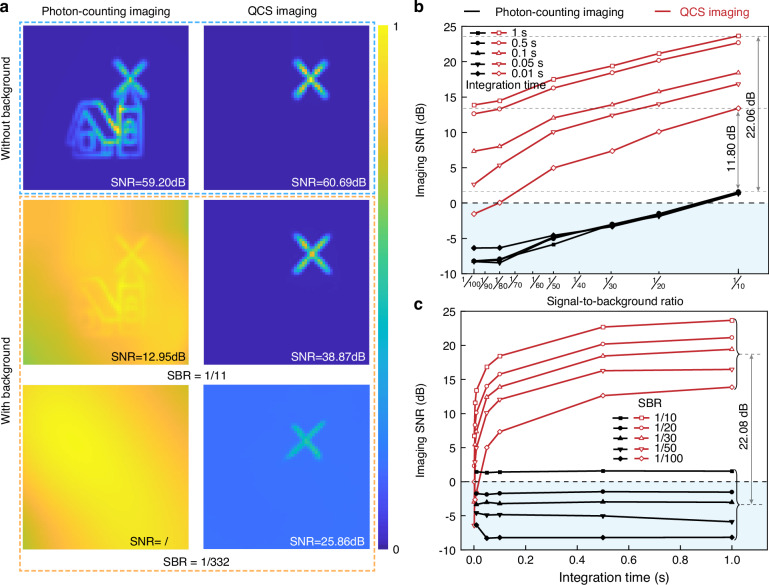
Investigation of the noise resistance of the QCS imaging. **a** Photon-counting imaging and QCS imaging with and without background noise. When there is no background noise, the SNR of photon-counting imaging and QCS imaging is 59.20 dB and 60.69 dB, respectively. Ideally, they should be the same, the difference is caused by an imperfect uniform distribution of light intensity. Notably, when the SBR is 1/11, QCS imaging exhibits a significantly higher SNR compared to photon-counting imaging by 25.92 dB. Even at an SBR of 1/332 where photon-counting imaging signals are completely submerged in noise, QCS imaging still maintains a relatively high SNR of 25.86 dB. The deep denoiser prior image restoration (DPIR) technique was used to optimize the image with background noise^45^ (see Supplementary Note 3 for the original image). **b** Imaging SNR versus SBR for integration times ranging from 0.01 s to 1 s. **c** Imaging SNR versus integration time for SBRs ranging from 1/10 to 1/100

When the object exhibits dynamic features, QCS imaging can effectively mitigate background noise interference by utilizing its broadband detection capabilities to extract the object’s dynamic features. To investigate the noise resistance capability of the QCS imaging system, a tungsten lamp (Thor labs, SLS202L, 450 nm to 5.5 μm) is used to simulate background noise. The intensity of the background noise is gradually increased through a continuously adjustable attenuator. The flicker frequency of the windmill is set at 1 MHz, and the photon count rate of the signal is set at 20 kcps. In general, background noise photons exhibit complete randomness without any dynamic characteristics. Therefore, extracting the dynamic features of the object with our imaging approach can significantly enhance noise resistance. Figure 3a present the imaging results of the photon-counting imaging and QCS imaging systems. The object becomes obscured by noise in photon-counting imaging when the SBR is set to 1/332; however, the QCS imaging system effectively reconstructs a clear windmill profile with a high SNR of 25.86 dB.

A comprehensive analysis of the crucial factors influencing the SNR, including the SBR and integration time, is presented in Fig. 3b, c. The SNR of the photon-counting imaging system sharply decreases with increasing background noise. When the SBR is less than 1/13, the SNR is less than 0 dB, indicating that the signal is indistinguishable from the noise; thus, no image information can be extracted. However, in the QCS imaging system, the SNR exceeds that of the photon-counting imaging system by 11.08–22.06 dB, as depicted in Fig. 3b. Moreover, the SNR of the QCS imaging system rapidly increases with increasing integration time and exceeds that of the photon-counting imaging system by 22.08 dB, as illustrated in Fig. 3c.

The SBRs mentioned in this study refer to the ratio of the detected photon count rate of the signal and background, without considering any other prior information about the object. Unlike spectral filtering, spatial filtering, and gating techniques used in active single-photon imaging, the method proposed in this paper provides strategies for passive single-photon imaging to accomplish all-weather tasks.

### Application of quantum compressed sensing

**Fig. 4:**
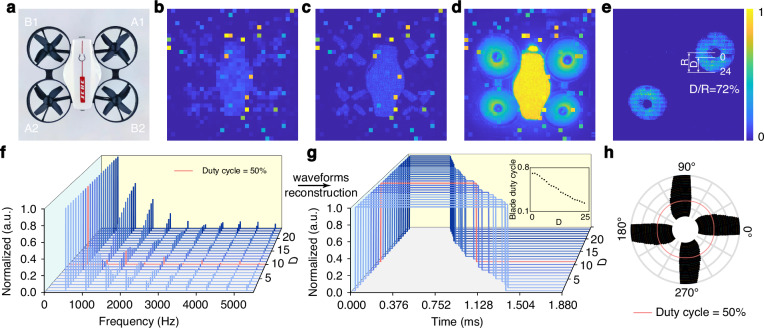
Drone rotor image reconstruction via QCS imaging. **a** A photograph of the drone. **b** Photon-counting imaging using the SPAD array. **c**, **d** Sub-pixel scanning photon-counting images of the drone in the “power off” and “power on” states, respectively. **e** QCS imaging at 530 Hz. R and D represent the overall radius and blade length of the drone rotor, respectively. **f** The spectrum of each pixel on blade length D in (**e**). **g** The time-domain waveforms reconstructed correspond to the spectra in (**f**). The inset shows the duty cycle of each waveform. **h** The reconstructed drone rotor pattern

To demonstrate the capability of QCS imaging in capturing broadband dynamic information within a realistic environment, we selected drone as our experimental object. A rotor drone is characterized by its compact size and dynamic properties due to the high-speed rotor rotation. The flight states of the drone were initially assessed in a controlled laboratory environment. The images were obtained using a 32 × 32 SPAD array^38–43^. Each pixel in the detector array operates independently, and the integrated time digital conversion circuit provides us with precise arrival times for each photon. By utilizing a signal reconstruction algorithm, rotor image of drone with sparse dynamic features could be recovered. The results are presented in Fig. 4, where Fig. 4a is a photograph of the drone and Fig. 4b shows direct 32 × 32 photon-counting images of the drone in the “power off” state. Figure 4c, d show photon-counting images of the drone in the “power off” and “power on” states with sub-pixel scanning imaging^44^. (See the Materials and methods for sub-pixel scanning imaging.) Limited by the long integration time of the photon-counting imaging system, real-time dynamic information about the rotor cannot be acquired when the drone is powered on. Figure 4e shows the dynamic imaging result of the drone captured by the QCS imaging system at 530 Hz, corresponding to the “hovering” flight state. Only two of the four rotors are shown here, and the other two have a characteristic frequency of 490 Hz. The stationary fuselage and other rotating rotors with different frequencies are significantly suppressed in the image. Each pixel in the image shows the dynamic feature at the corresponding spatial position.

In this study, the signal *x* represents the modulation waveform of the radiated light field generated by the rotation of the drone rotor. The signal reconstruction algorithm of the QCS system estimates the signal *x* by employing a time series *y* = [*y*_1_, *y*_2_, …, *y*_*m*_, …*y*_*M*_] of photon detections, as described in Eq. (3). Firstly, a sparse representation of the signal in the frequency domain was obtained by performing discrete Fourier transform on the time series, as shown in Fig. 4f. It illustrates the spectrum of each pixel along the blade length D in Fig. 4e, which includes nonzero terms ranging from the fundamental frequency to the tenth-order frequency. Then the estimation sparse coefficients were extracted from Fig. 4f by setting a threshold. The time-domain waveform of signal of each pixel can be reconstructed using the sparse coefficients according to Eq. (3), as shown in Fig. 4g. Each rotor is equipped with four blades, resulting in the presence of four square waves during each rotation. To show the waveform more clearly, only a quarter-period waveform is presented in Fig. 4g. The peak and trough of the recovered square wave represent the blade and non-blade areas of the drone, respectively. Spanning from the center to the edge of the rotor, the duty cycle of the time domain waveform varies from 73.4% to 22.3%, where the pink curves depicted in Fig. 4f–h represent demarcation lines with a 50% duty cycle. (In Supplementary Note 4, the rotor reconstruction of another drone with two blades is discussed.) The position of the fan blade is determined according to the ratio of R and D in Fig. 4e. The rotor pattern of the drone can be reconstructed by plotting these data in polar coordinates, as shown in Fig. 4h.

The image is a binary representation (0, 1) obtained by applying a threshold of the half-height width to the normalized time-domain waveform. See Supplementary Note 2 for detailed signal reconstruction algorithm of the QCS imaging. In principle, according to the results of QCS imaging bandwidth measurement in Fig. 2d, our work enables the reconstruction of images with rotational speeds exceeding GHz level. This breakthrough overcomes the frame rate limitation inherent in conventional imaging technology, thereby offering a novel approach for achieving high-quality broadband imaging.

**Fig. 5:**
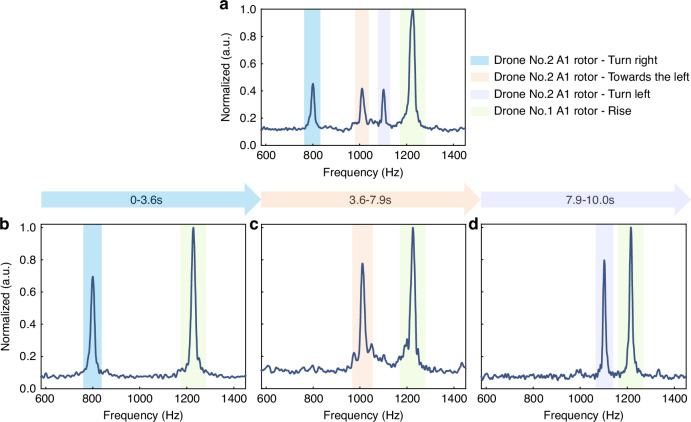
Drone flight states captured in real time. **a** The characteristic spectrum of the two drones. **b**–**d** The characteristic spectra across distinct temporal periods

The QCS imaging provides us a new method for detecting drones by extracting their dynamic features. To demonstrate this, we first systematically characterize the dynamic features of the drone. The drone utilized for experiments is equipped with four rotors and has eight flight states. We investigated the characteristic frequencies of these four rotors across the eight flight states using different drone models. The four rotors can be classified into two distinct groups, namely, A and B (Fig. 4a), with each group consisting of two diagonally positioned rotors. The characteristic frequences of the rotors are directly proportional to the rotational speed. Table 1 lists the characteristic frequencies for the eight flight states of Drone No. 1 (Shantou Xinkaiyang Toy Technology Co., Ltd., mini 905). It shows that in the “hovering” state of the drone, all four rotors stabilize at a lower speed ~531 Hz. As the drone transitions to the “rise” state, the rotational speed rapidly increases to ~1239 Hz. We compared the characteristic frequencies of the flight states for three different drone models, showing that each model of drone exhibits distinct characteristic frequencies. Therefore, the flight state and drone model can be accurately identified by extracting characteristic frequencies from a QCS image of a moving drone. To assess the efficacy of QCS imaging in drone identification, the characteristic frequencies of two models of drones were measured in real time during different flight states, as depicted in Fig. 5. Drone No. 1 maintained a “rise” flight state throughout the test, while the flight state of changed over time (see Supplementary Note 5 for the characteristic frequencies of Drone No. 2). The spectrum, shown in Fig. 5a, exhibits four characteristic frequencies that were captured during a 10-second integration time. In the time segment spectrum, we observed changes of the drone’s flight states over time, as show in Fig. 5b–d. The characteristic frequencies captured at intervals of 0–3.6 s, 3.6–7.9 s, and 7.9–10 s are (1228 ± 5, 800 ± 4), (1228 ± 5,1010 ± 5), and (1228 ± 5,1103 ± 3) respectively, corresponding to the flight states of Drones No. 1 and No. 2 (rise, turn right), (rise, towards the left) and (rise, turn left).

**Table 1:** The characteristic frequencies for the eight flight states of Drone No. 1

Drone fight states	A1 rotor/Hz	A2 rotor/Hz	B1 rotor/Hz	B2 rotor/Hz
Hovering	525 ± 4	536 ± 3	529 ± 2	533 ± 3
Rise	1234 ± 28	1219 ± 20	1271 ± 9	1233 ± 12
Turn left	1229 ± 3	1321 ± 14	773 ± 5	718 ± 4
Turn right	786 ± 6	707 ± 7	1255 ± 8	1226 ± 4
Move forwards	988 ± 23	811 ± 13	1196 ± 12	1194 ± 6
Back	743 ± 29	936 ± 11	1092 ± 4	1285 ± 3
Towards the left	926 ± 13	789 ± 28	1081 ± 3	1247 ± 4
Towards the right	779 ± 10	996 ± 5	1187 ± 6	1131 ± 5

### 10 km drone detection with QCS imaging

**Fig. 6:**
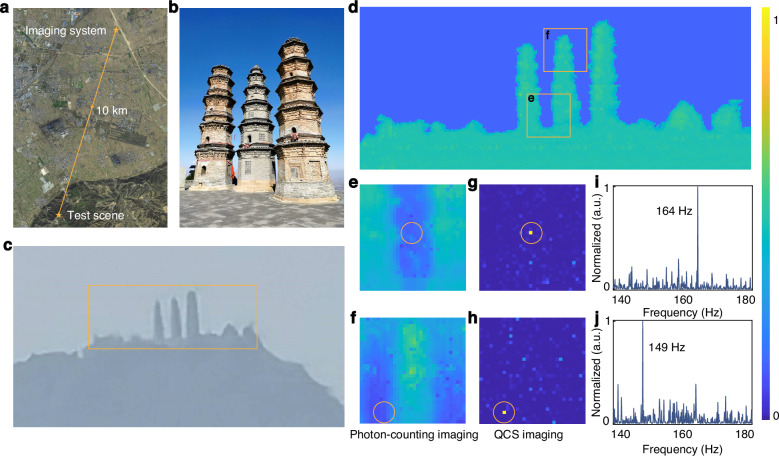
10-km passive drone detection using QCS imaging. **a** The satellite image of the test scene, in which the experimental system is placed in Taigu East Station, and the drone is located in Phoenix Mountain, Taigu District, Shanxi Province. **b** A close-up image of the test scene. **c** The photo of the scene taken by a digital camera at a distance of 10 km. **d** The photon-counting imaging of the three towers of Phoenix Mountain. **e**, **f** The photon-counting imaging of the two regions where the drones are located. **g**, **h** The corresponding QCS imaging of (**e**) and (**f**). **i**, **j** The dynamic characteristic spectrums of the drones

The drone appears as a point object within the imaging field of view when it is flying at a considerable distance. As a point object, the drone cannot be identified directly with traditional passive imaging technology. The imaging technique discussed in this paper is a passive imaging method. Here, we conducted daytime observations under natural lighting conditions without additional lighting sources. QCS imaging technology is used to capture the sunlight scattered by the drone’s rotor and extract the dynamic features of the drone at the single-photon level to achieve accurate point target recognition. Field tests were performed in Phoenix Mountain, located in Taigu District, Shanxi Province. The imaging system is located at Taigu East Station, with a test distance of 10 km, as shown in Fig. 6a, b shows a close-up photo of the object imaging scene, and Fig. 6c displays a photo taken by a digital camera (Nikon D5600) from 10 km. During the test, the weather condition was clear, and the longitude and latitude coordinates of the observation point were E: 112.617723°, N: 37.446663°. The longitude and latitude coordinates of the drone take-off point are E: 112.585449°, N: 37.362617°. The detector used in the experiment is an SPAD array with a scale of 32 × 32 pixels, and each pixel is operated in time-correlated single-photon counting mode, see the Materials and methods for a detailed description of the experimental system. The experiment was carried out during the daytime, and a PTZ was used for scanning imaging. Figure 6d shows the photon-counting imaging of the scene in Fig. 6c. The outlines of the three towers and the mountain can be distinguished, but the drone cannot be. The two Drone No.4 (DJI Marvic 3) are located in Fig. 6e, f, respectively. They occupy only 1 or 2 pixels in the images. Drone No. 4 lacks a rotor guard, unlike the drone depicted in Fig. 4, allowing for the capture of dynamic rotor information from any perspective. The imaging system’s altitude, as measured by a GPS surveyor, is 800 meters, while Drone No. 4’s take-off altitude is 1214 meters. Taking into account the altitude difference and the drone’s hovering height, the calculated viewing angle is 2.4°. Although the detection SNR is lower compared to top or upward views, it is still feasible to capture the dynamic characteristics of the drone’s rotor (refer to Supplementary Note 9 for different perspectives on observing drone rotors). QCS imaging can extract the dynamic characteristics of the drone’s rotor to suppress the background noise. Figure 6g, h show QCS imaging, and the position of the drone can be easily determined. Figure 6i, j show the corresponding drone characteristic spectrum, with characteristic frequencies of 164 Hz and 149 Hz, respectively. The characteristic frequencies of different types of drones are influenced by factors such as their volume, weight, the number of blades on the rotor, and the performance of the rotor motor, leading to significant variations. Specifically, Drone No.4 features a two-bladed rotor, larger overall dimensions but with a greater rotor area, stronger materials, and more powerful rotor motors. Consequently, in the hovering state, Drone No.4 exhibits a lower characteristic frequency compared to Drones No.1–3. The characteristic frequencies for various flight states of the drones are detailed in Table S3 of Supplementary Note 5. The difference in the characteristic frequencies here is mainly due to wind. In addition, we conducted a QCS large-field high-resolution single-photon imaging of Twin Pagoda Temple at a distance of 400 m, and the experimental results are shown in Supplementary Notes 6–8.

Although we have listed the characteristic frequencies of different flight states of drones and experimentally verified the feasibility of drone identification. However, in practical applications, identifying the model and flight state of an unknown drone still requires a significant amount of work, such as building a database of characteristic frequencies for all drone models. Furthermore, the characteristic frequency of drones is subject to interference from various factors, such as drone battery capacity, drone payload, altitude, and weather conditions. So, we believe that in real-world applications, it may only be possible to identify the type of drone, such as toy drones, professional-grade aerial photography drones, payload drones, and racing drones, given that the rotor speed of these types of drones varies significantly. This effectively addresses the application requirements for the majority of drone detection.

## Discussion

In this work, a broadband QCS imaging method is proposed and used for remote passive drone detection. It achieves the direct capture of broadband dynamic features based on discrete sparse photon detections. The detectable bandwidth reaches up to 2.05 GHz, which is six orders of magnitude higher than the most advanced photon-counting imaging technology. The maximum detectable rotational speed is determined by the time jitter of photon detection. Additionally, this method demonstrates excellent noise resistance by extracting dynamic features of the objects. High-quality imaging was realized even with a signal-to-background ratio of 1/332. Since the rotor of the drone exhibits obvious dynamic characteristics during flight, it provides a solution to the long-standing problem of remote drone detection. We successfully demonstrated drone detection at distances up to 10 km. The technique also holds promise for high-speed imaging, such as engine speed monitoring in aerodynamic experiments and non-contact vibration imaging in industrial detection, advancing the practical use of single-photon imaging in real-world scenarios.

## Materials and methods

### Experimental set-up for QCS imaging

**Fig. 7:**
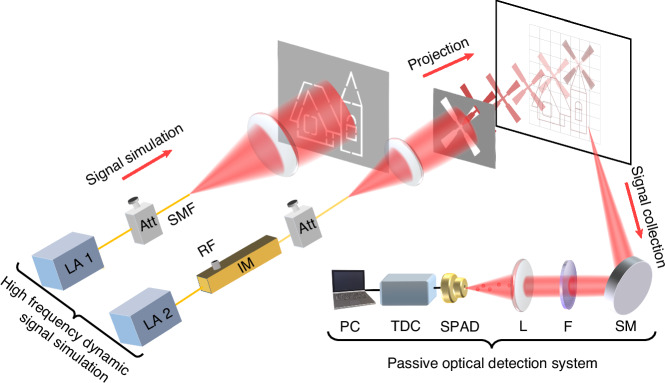
Schematic diagram of simulated windmill house experiment. The windmill part of the pattern has dynamic characteristics, with an adjustable flicker frequency ranging from 10 kHz to 3.0 GHz, while the house part remains static. LA laser, Att attenuator, IM intensity modulation, SMF single-mode fiber, RF radio frequency, SM scanning mirror, F filter, L lens, SPAD single-photon avalanche detector, TDC time-to-digital converter, PC personal computer

To quantitatively evaluate the bandwidth and noise resistance of QCS imaging, an experimental setup with controllable bandwidth and noise was established. The experimental setup consists of an object simulation module and a signal detection module, as depicted in Fig. 7. The object simulation module is used to simulate the object with high-frequency dynamic characteristics. This module consists of two distributed feedback laser (DFB) lasers (Sichuan lightsource optoelectronic technology Co. LTD., DFB-1550-10-PM-FA-M), with one laser beam modulated by an electro-optical modulator to generate the windmill section characterized by a high-frequency flicker, and the other beam utilized to generate the remaining static portion. The two beams are attenuated and projected onto a screen, resulting in the formation of a high-frequency dynamic pattern. This pattern incorporates windmill blades and houses, with the windmill blades projected using the modulated laser. To replicate the background noise present in real-world environments, a light source is used to illuminate the imaging area. The signal detection module is utilized for photon collection and detection. A scanning mirror is used to scan each point in the projected pattern. Photon detection is carried out using an SPAD, and photon clicks are recorded using a TDC.

### Sub-pixel scanning imaging

The imager (Photon Force, PF32) used in the experiment integrates 32 × 32 SPADs, however, it still not enough to directly take a clear image. Therefore, it is necessary to expand the size of pixels to improve the image quality. In the experiment, a method of sub-pixel scanning was used for imaging. We developed a programmable controller based on a single-chip microcomputer to control the high-precision scanning mirror (Newport, FSM-300-01) in order to achieve sub-pixel scanning imaging using the SPAD array. Considering the pixel pitch (50 μm) and SPAD active area (diameter = 6.95 μm), sub-pixel division factors was set to 7. A high-resolution image is obtained through data post-processing.

### Experimental system for drone field detection

To demonstrate the remote drone detection, a Schmidt-Cassegrain telescope (Celestron, AVX925, f = 2000 mm, D = 200 mm) was used as the camera lens. The signal light passes through the scanning mirror and is divided into two beams by a beam splitter. One is recorded by the SPAD array detector for QCS imaging, while the other one is captured by a CCD (Thor labs, CS165CU/M) for field of view calibration at the beginning of the experiment. The imaging system is mounted on a heavy-duty PTZ (Shandong Feiyue Electronics Technology Co., Ltd, SP5050), with a scanning step of 0.01°. The PTZ and scanning mirror constitute the scanning imaging system, where the PTZ performs coarse scanning for a wide field of view, while the scanning mirror performs sub-pixel fine-scanning imaging. By developing a programmable cooperative control module based on a single-chip microcomputer to control the PTZ and scanning mirror, single-photon imaging of wide-field scenes could be achieved.

## Supplementary information

Supplementary Information for 10-km passive drone detection using broadband quantum compressed sensing imaging

Source data

## Data Availability

The data that support the plots and tables within this paper and other findings of this study are available from the corresponding authors upon reasonable request.
