# Supplementary material for: 10-km passive drone detection using broadband quantum compressed sensing imaging
Source: Light Sci Appl. 2025 Jul 14;14:244. doi: 10.1038/s41377-025-01878-y (PMC12259967; doi:10.1038/s41377-025-01878-y)
Supplement: Supplementary file 1 — Supplementary Information for 10-km passive drone detection using broadband quantum compressed sensing imaging [file 41377_2025_1878_MOESM1_ESM.docx]

Supplementary Information for

**10-km passive drone detection using broadband quantum compressed sensing imaging**

Shuxiao Wu^1,2,#^, Jianyong Hu^1,2,#^, Jiaqing Ge^1,2^, Yanshan Fan^1,2^, Zhexin Li^1,2^, Liu Yang^1,2^, Kai Song^3^, Jiazhao Tian^3^, Zhixing Qiao^4^, Guosheng Feng^4^, Xilong Liang^5^, Changgang Yang^1,2^, Ruiyun Chen^1,2^, Chengbing Qin^1,2^, Guofeng Zhang^1,2^, Liantuan Xiao^1,2,3*^ and Suotang Jia^1,2^

*^1^State Key Laboratory of Quantum Optics Technologies and Devices, Institute of Laser Spectroscopy, Shanxi University, Taiyuan, 030006, China.*

*^2^Collaborative Innovation Center of Extreme Optics, Shanxi University, Taiyuan, 030006, China.*

*^3^College of Physics,* *Taiyuan University of Technology, Taiyuan, 030600, China.*

*^4^College of Medical Imaging, Shanxi Medical University, Taiyuan, 030001, China.*

*^5^Department of Materials and Chemical Engineering, Taiyuan University, Taiyuan, China*

*^#^These authors contributed equally: Shuxiao Wu, Jianyong Hu.*

**Corresponding author E-mail address: jyhu@sxu.edu.cn; xlt@sxu.edu.cn*

**Table of contents**

**Supplementary Note 1. Theoretical model of quantum compressed sensing**

**Supplementary Note 2. QCS image reconstruction algorithm**

**Supplementary Note 3. Imaging with the Deep Denoiser Prior Image Restoration Technique**

**Supplementary Note 4. Rotor image reconstruction by the QCS imaging system**

**Supplementary Note 5. The characteristic frequencies of Drones No. 2, No. 3 and No. 4**

**Supplementary Note 6. QCS imaging of drone**

**Supplementary Note 7. Field test of QCS imaging**

**Supplementary Note 8. Photograph of the Twin Pagodas Temple**

**Supplementary Note 9.** **Different** **perspectives for observing drone rotors**

**Supplementary Note 1. Theoretical model of quantum compressed sensing**

In the main text, quantum compressed sensing (QCS) is used to directly capture the dynamic information of the target, resulting in significant improvements in imaging bandwidth and data compression rate compared to existing technologies. QCS uses the quantum behaviour of photons to construct a passive-adaptive compression measurement model, which is fundamentally distinct from the non-adaptive measurement method of classical compressed sensing (CS) where the measurement matrix is predetermined artificially. Here we illustrate the quantum advantage of QCS by comparing CS with QCS. Considering the logic of the statement, we first provide a brief review of classical CS.

**Mathematical model of classical CS**

CS is a signal processing technique for efficiently acquiring and reconstructing a sparse signal, by finding solutions to underdetermined linear systems. The sparsity of a signal can be exploited to recover it from far fewer samples than required by the Nyquist-Shannon sampling theorem. There are two steps for CS, **signal sampling and reconstruction.**

Here we take random sampling (also known as non-uniform sampling) CS as an example, which works by taking fewer non-adaptive random measurements. The CS sampling model can be mathematically described by:

 (S1)

where, *x*∈ℝ*^N^* is an input signal of length *N*, *φ*∈ℝ*^M^*^×^*^N^* is an *M*×*N* random measurement matrix, *y*∈ℝ*^M^* is the measurement vector of length *M*. The input signal and the random measurement matrix are multiplied together to generate compressive measurements. Here, the number of measurements taken are much lesser than the length of input signal, i.e., *M*<<*N*. Since the measurement matrix is a predetermined random matrix and fixed independently of signal *x*, this measurement method is **non-adaptive**^1^.

The signal reconstruction of CS involves recovering the original signal *x* from the measurement vector *y* by solving the equation *y*=*φx*, which is an underdetermined system of linear equations and have infinite number of possible solutions. Since the signal *x* is sparse in some transform domain. It can be represented as a linear combination of the sparsifying basis and the sparse coefficients:

$x=\sum_{i=1}^{N} s_{i}\Psi_{i}=\Psi s$ (S2)

where, *s*∈ℝ*^N^* is the sparse coefficients vector of length *N*, having *k* (*k*<*M*<<*N*) significant/nonzero terms. Therefore, the key to signal reconstruction is to solve the sparse coefficients *s*. In such cases, the unique solution can be obtained by posing the reconstruction problem as an *ℓ*_0_-optimization problem:

$\hat{s}=\text{arg}\min_{s} \left\|s\right\|_{0}\text{ subject to, }As=y$ (S3)

where *ŝ* is an estimation of *s* and ||*s*||_0_ denotes the *ℓ*_0_-norm of *s*, which represents the number of significant/nonzero elements. The inputs to the reconstruction algorithm are the measurement vector *y* and reconstruction matrix *A*=*φΨ*. The output of CS reconstruction algorithm is an estimation of sparse representation of *x*, i.e., *ŝ*. The estimation of *x*, i.e., $\hat{x}$ can be obtained from *ŝ* by taking its inverse transform ^2^.

**Mathematical model of QCS**

QCS is a signal processing technique for efficiently acquiring and reconstructing a sparse signal from considerably fewer samples than required by the Nyquist-Shannon theorem by constructing a compressive measurement system using quantum resources, such as quantum coherence, quantum entanglement, etc. Similar to classical CS, QCS also requires the input signal *x* to have sparsity in some transform domain. The difference is that the classical CS implementation process is divided into two steps: signal sampling and reconstruction, while QCS requires four steps: initial quantum state preparation, quantum state manipulation and detection, and signal reconstruction. Moreover, classical CS is a non-adaptive measurement method, whereas QCS is a passive-adaptive measurement method that exhibits evident quantum advantages.

**The initially prepared quantum state** |*ψ*〉 can be either pure or mixed states. The requirement is that they should follow a uniform distribution in the original or some transformed domain when measured directly. In this work, signal *x* is a time-varying signal with sparsity in the frequency domain. As a passive imaging system, the signal is ambient scattered light and is a mixed state, which can be represented by density operators:

$\rho=\sum_{i=1}^{N} p_{i}|\psi_{i}\rangle\langle\psi_{i}|$ (S4)

*p_i_* = 1/*N* represents the fraction of the ensemble in each pure state |*ψ_i_*〉, which satisfies the normalization condition. If the initial quantum state is directly measured, the probability of the photon measurement collapsing to any time is equal, resulting in a white noise distribution in the sparse domain of the signal (i.e. the frequency domain), where each frequency component carries equal weight.

**Quantum state manipulation** involves mapping the signal *x* to the initial quantum state in order to ensure that the measured collapse probability distribution aligns with the signal waveform ^3-5^, while preserving the sparsity of the signal in the transform domain. In this work, the radiation signal of the target has in fact been manipulated, that is:

$\rho=\sum_{i=1}^{N} x_{\left(t\right)}p_{i}|\psi_{i}\rangle\langle\psi_{i}|$ (S5)

The signal with frequency domain sparsity can be decomposed into sine waves with different fundamental frequencies using Fourier series:

 (S6)

where *ϕ* is the initial phase, *k* (*k*<*M*<<*N*) represents the number of significant/nonzero elements.

**Quantum state detection** is the sampling step of QCS. The mathematical model of QCS can generally be described as follows:

$y=Â|\psi^{x}\rangle$ (S7)

where |*ψ*〉 represents the initial quantum state, *x* denotes the signal to be measured, *Â* denotes the measurement operator, and *y* represents the measurement result. The quantum state |*ψ^x^*〉 corresponds to the process of manipulating quantum state |*ψ*〉 with signal *x*. Then, measurement operator *Â* acts on quantum state |*ψ^x^*〉, corresponding to the process of quantum state detection. In this work, the quantum state to be detected is a mixed state, and the detection process can be described as follows:

$\begin{matrix} \langle n\rangle=\mathrm{Tr}\left(\rho\hat{n}\right) \\ \begin{matrix} & \begin{matrix} =\mathrm{Tr}\left(\sum_{i=1}^{N} x_{\left(t\right)}p_{i}|\psi_{i}\rangle\langle\psi_{i}|\hat{n}\right) \\ =\sum_{i=1}^{N} x_{\left(t\right)}p_{i}\langle\psi_{i}|\hat{n}|\psi_{i}\rangle\\ =\sum_{i=1}^{N} x_{\left(t\right)}p_{i}\left\langle\hat{n}\right\rangle_{i} \end{matrix} \end{matrix} \end{matrix}$ (S8)

It shows that the probability of photon detection is directly related to the signal waveform. The result of the detection is a vector *y*={*y*_1_, *y*_2_, ···, *y_m_*, ···, *y_M_*, *M*<<*N*} of length *M*, representing the photon arrival time series. Unlike the non-adaptive sampling mode used in classical CS, the photon measurement collapse in QCS is directly related to the signal, and the unknown non-cooperative signal can be automatically matched, which is a **passive-adaptive measurement** ^1^.

**Signal reconstruction** of QCS is similar to classical CS. Since the target signal in this work is sparse in the frequency domain. According to Eq. S6, the key to signal reconstruction lies in obtaining the non-zero sparse coefficients:

$\hat{s}_{k}=\sum_{j=1}^{M} \exp\left(2\pi f_{k}y_{j}\right)$ (S9)

where *y_j_* is the detected photon arrival time and *M* is the photon counts; *ŝ* is the estimation of non-zero elements of sparse coefficients. $\hat{x}$ can be obtained from *ŝ* by taking its inverse transform:

$\hat{x}=\Psi^{-1}S$ (S10)

where *Ψ* is the sparsifying basis of the signal *x*, *S* is set of sparse coefficients of length *k*.

**A simulation example** is given to make a more intuitive comparison between classical CS and QCS. Considering the periodic Gaussian pulse as the input signal, we set the pulse repetition frequency to 50 kHz and full width at half maximum (FWHM) to 10^-6^ s. For an unknown signal, the optimal strategy of classical CS technology is to sample the signal randomly. To ensure that the simulation results are not affected by device selection, we assume here that the analog-to-digital converter used has an ideal time resolution of 10^-12^ s and an amplitude resolution of 10^-4^ V. However, due to the relatively small duty cycle of the signal, most of the sampling is distributed in the position of amplitude zero or close to zero, as shown in Fig. S1(a). According to the Fourier transform, these samples of zero amplitude do not contribute to the spectrum analysis. Therefore, most of the sampling points of classical random sampling CS technology are invalid sampling. In contrast, in QCS, the detection probability of a photon is directly related to the waveform amplitude. If the signal amplitude is zero or close to zero, the probability of the photon collapsing to that moment is also zero or close to zero. This passive-adaptive sampling automatically collapses into the non-zero region of the signal, significantly improving sampling effectiveness. This can be seen intuitively from the statistical distribution of Fig. S1(a) and S1(d). We investigated the sparse coefficients recovery of both methods when there is consistency between the random sampling number in classical CS and the photon count in QCS. The results show that the estimated results of sparse coefficients by QCS are closer to the true value, as shown in Fig. S1(b) and S1(e), where the black dotted line is the reference of the ideal results. This also leads to high-frequency noise in the reconstructed signal of classical CS, Coefficient of determination (*R*^2^) of Fig. S1(c), S1(f) and the original waveform were 0.4306 and 0.9514, respectively.

**
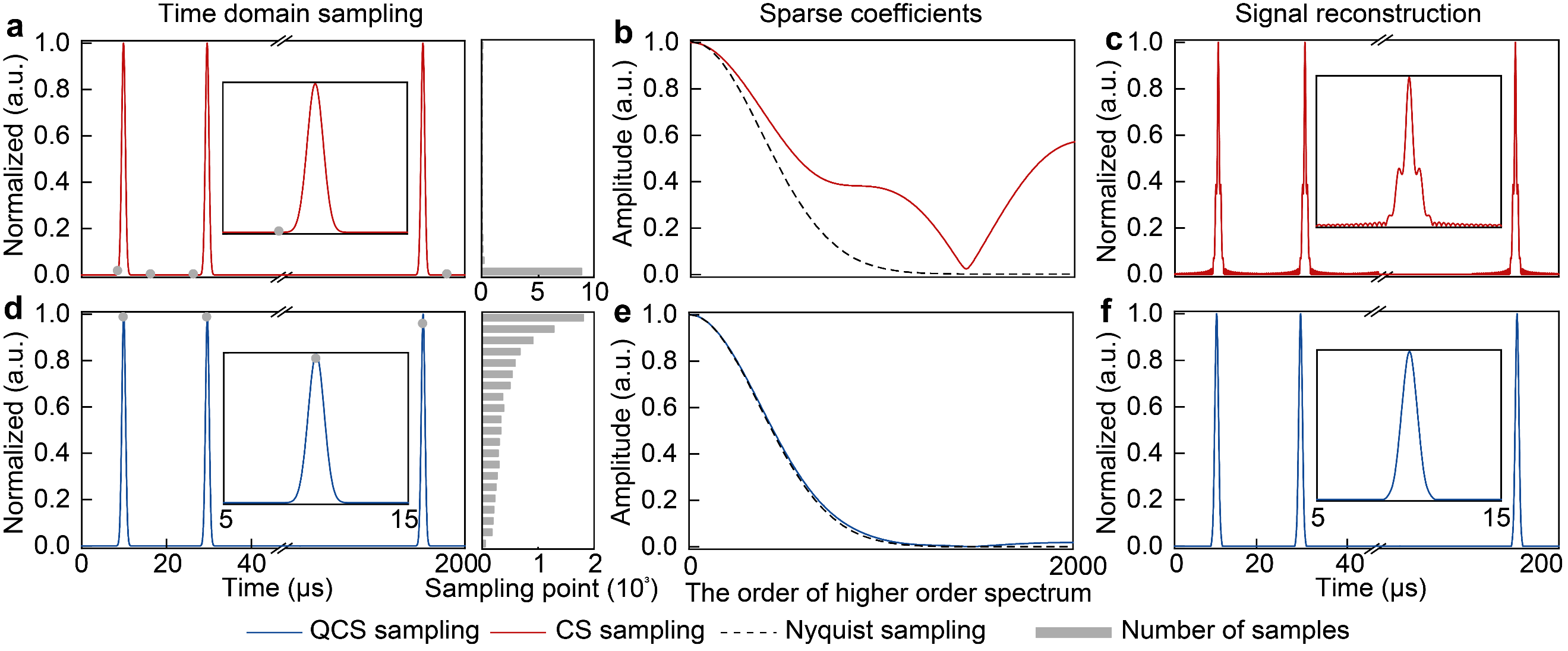
**

**Fig. S1 A simulation comparison of classical CS and QCS. a,** A periodic Gaussian pulse signal with random sampling of classical CS, the sampling rate is 100 Hz. **b,** Sparse coefficients recovered by random sampling based on classical CS, where the black dotted line is the true value. **c,** Signal reconstructed based on classical CS. **d,** A periodic Gaussian pulse signal with QCS sampling, the photon count rate is 100 cps. The inset in (**a**) and (**d**) are the distribution of signal amplitudes corresponding to classical CS and QCS sampling points respectively. It can be seen that QCS sampling points are distributed at positions where the signal amplitude is not zero, so that each sampling point contributes to the signal reconstruction. **e,** Sparse coefficients recovered by QCS sampling, where the black dotted line is the true value. **f,** Signal reconstructed by QCS.

**Supplementary Note 2. QCS image reconstruction algorithm**

In order to better understand the QCS imaging process. Fig. S2 shows a schematic diagram of the data acquisition and reconstruction algorithm of the QCS imaging system. According to the QCS imaging theory described in the main text, this process can be divided into four parts: initial quantum state preparation, quantum state manipulation, quantum state detection and signal reconstruction. In the initial quantum state preparation part, the drone rotor in the “power on” state is the measured signal *x*. During the QCS sampling process, the dynamic photons generated by the high-speed rotation of the rotor are measured to obtain the initial quantum state |*ψ*〉. In the process of quantum state manipulation, the measured signal *x* is used to manipulate the initial quantum state to obtain |*ψ^x^*〉, and its density operator is *ρ*. By measuring the effect of operator *Â* on the quantum state, the quantum state measurement result *y* is obtained. Based on the QCS imaging results, an appropriate rotor area is selected, and the pixels in this area are analysed individually to extract nonzero values. The sparse coefficients are the normalized amplitude of the non-zero frequency component of the spectrum, as shown in Fig. 4(f) and Fig. S5(f). We exploit the sparse nonzero values for waveform reconstruction. Fig. 4 and Fig. S5 show the rotor recovery results of four rotors and two rotors drones, respectively, which verify the universality of this method. By setting a threshold to optimize the waveform and determining the proportion of the rotor relative to the overall drone in the QCS image, the duty cycle of the rotor at different pixel positions is obtained. Finally, in the signal reconstruction part, the inverse Fourier transform algorithm is used to plot the duty cycle in polar coordinates to obtain the restored rotor image.

**
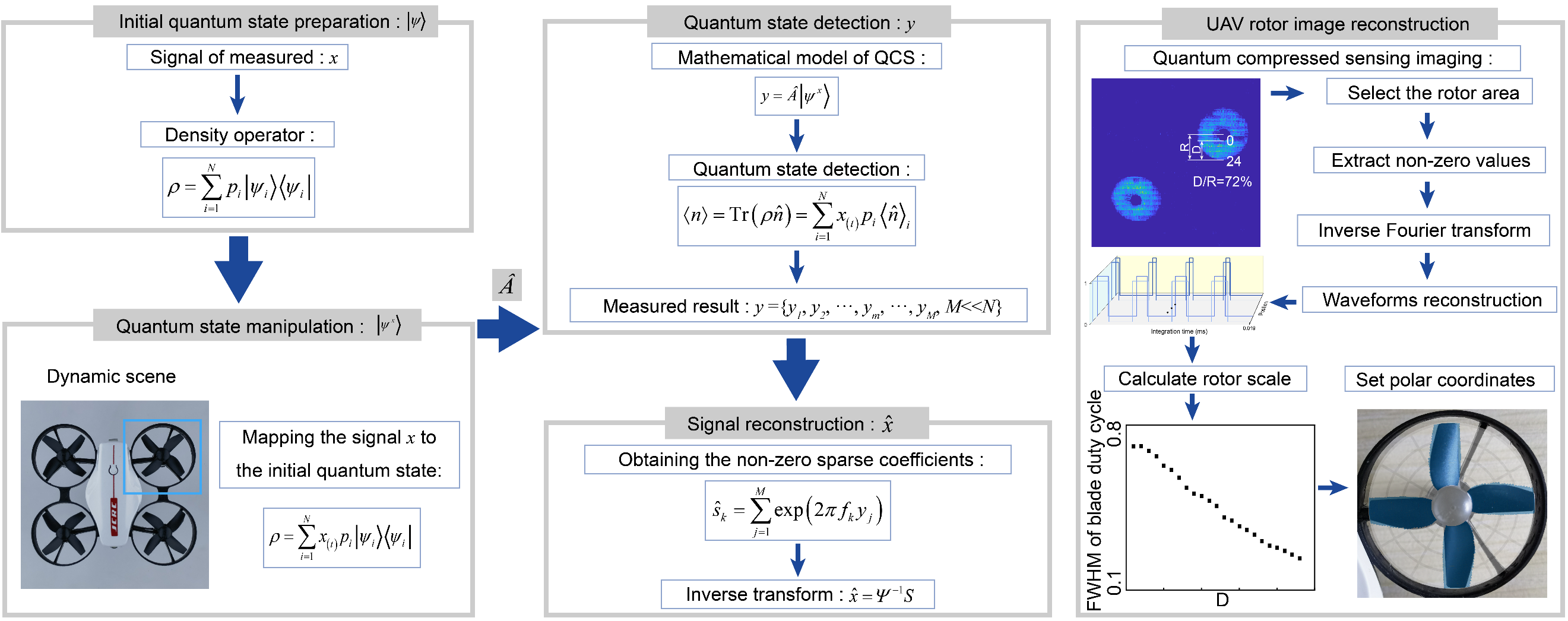
**

**Fig. S2 Schematic diagram of the image restoration algorithm.**

**Supplementary Note 3. Imaging with the Deep Denoiser Prior Image Restoration Technique**

The deep denoising prior image restoration (DPIR) is a flexible and effective deep denoisers for plug-and-play image optimization^6^. It has excellent performance in the applications of image deblurring, super-resolution and color image demosaicing. In this work, DPIR was used to optimize the image. In Fig. 3(a) of the main text, we present the DPIR optimization image, while Fig. S3 shows the corresponding original image.

**
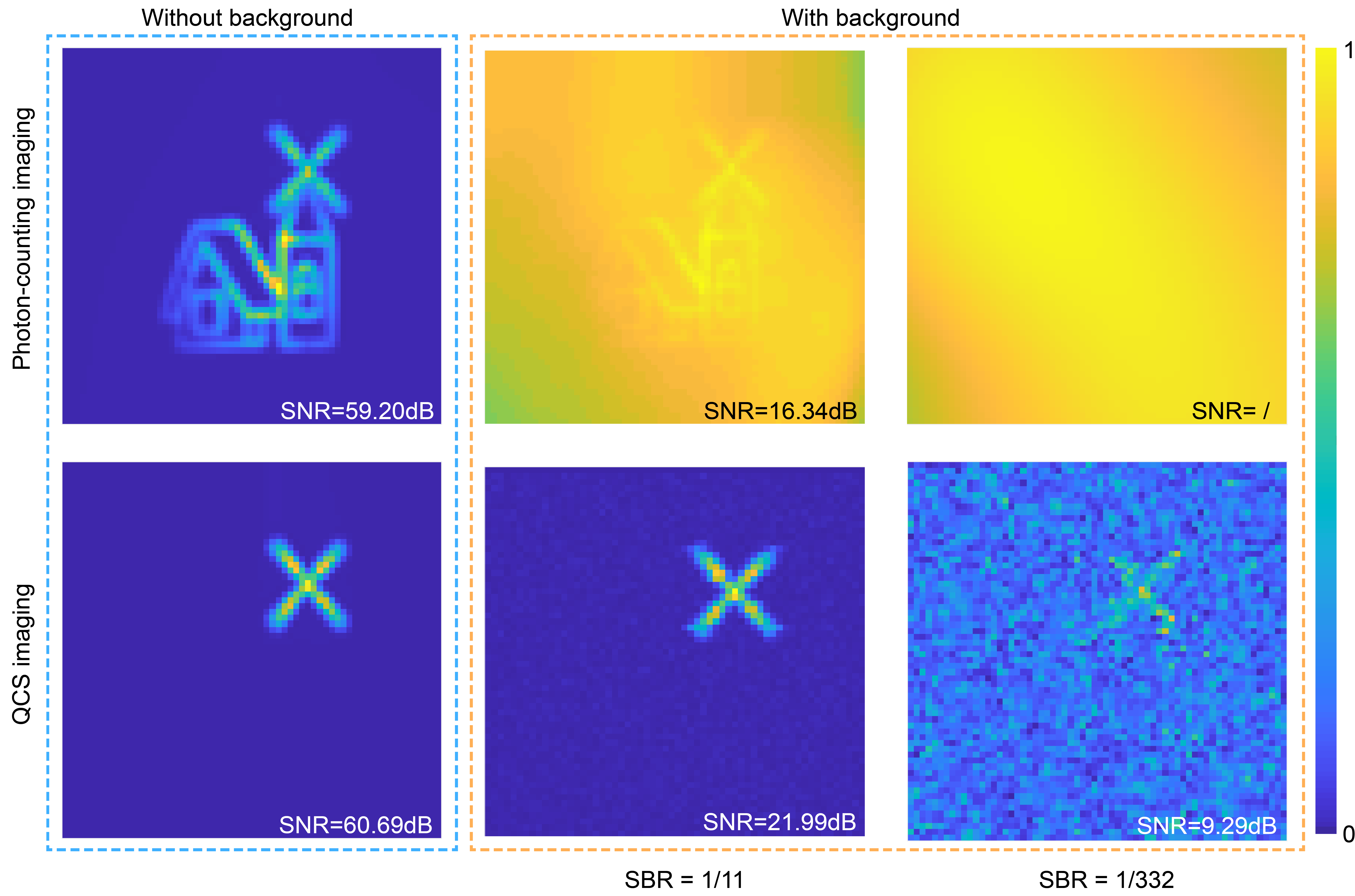
**

**Fig. S3 QCS image with background noise.** Original photon-counting imaging and QCS imaging results with and without background noise. All the images were obtained without DPIR optimization.

In the main text, we present the imaging results for signal-to-background ratio (SBR) of 1/11 and 1/332. Fig. S4 shows additional data, with Fig. S4(a) showing the photon-counting imaging results and Fig. S4(b) showing the QCS imaging results. The results for six different SBR values ranging from 1/11 to 1/332 are shown, along with the DPIR optimization results. In the experiment, the noise was introduced by controlling the intensity of the tungsten lamp with an adjustable attenuator. Since the noise photon counts is larger than the signal photon counts and difficult to control accurately, the SBR value in Fig. S4 is calculated based on experimental data. For the photon-counting imaging system, when the SBR is 1/11, a portion of the target image profile is visible despite the high background noise. However, after undergoing DPIR optimization, the signal is considered as noise and eliminated, resulting in only background noise remaining. In the other cases, the photon-counting imaging system cannot discern the target due to the significant background noise. Conversely, even at an SBR of 1/332, the contour of the target remains visible in the QCS imaging results. Furthermore, following DPIR optimization, the image quality is substantially enhanced, and the SNR is improved by 12.43-16.88 dB.

**
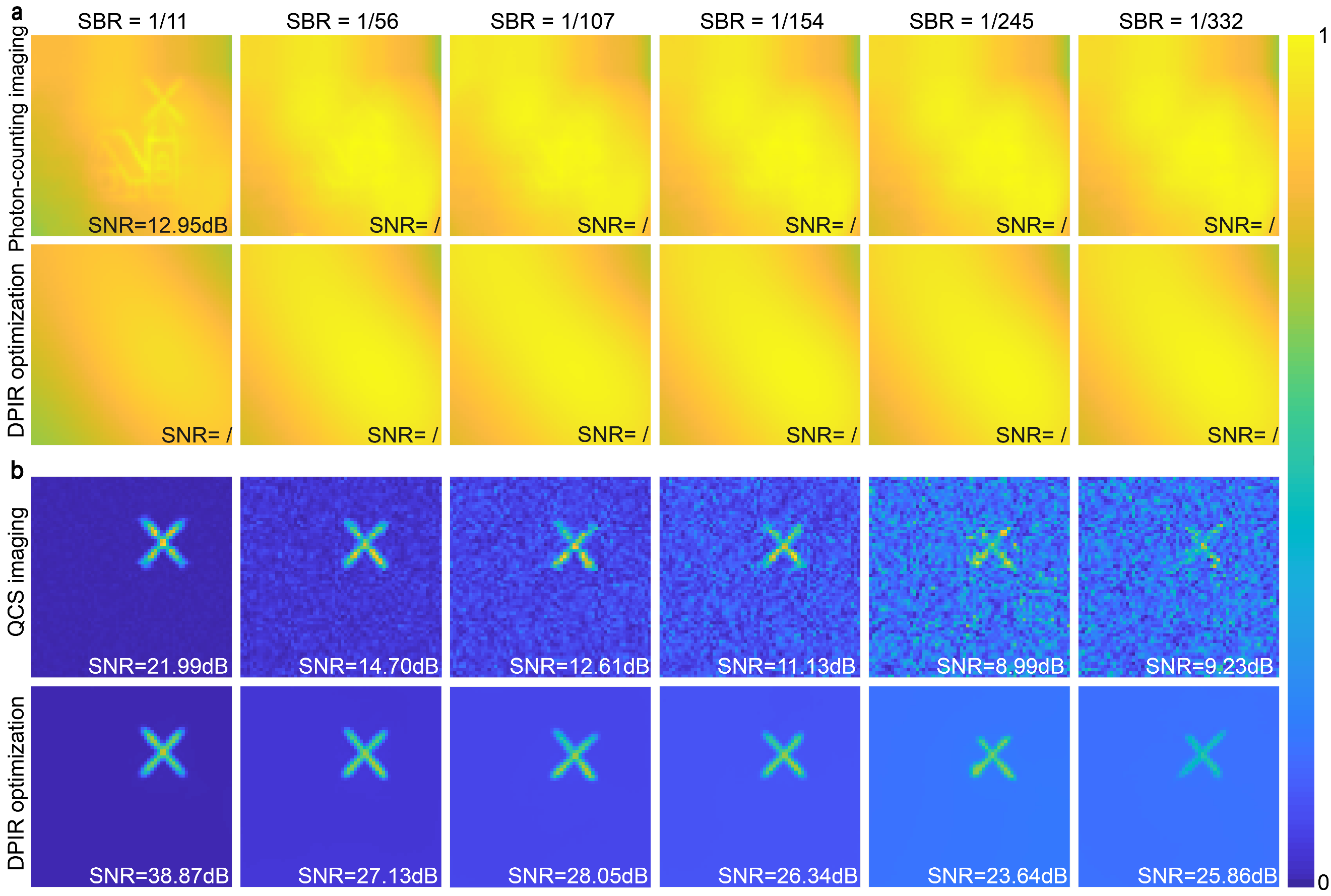
**

**Fig. S4 DPIR optimization of QCS images with strong background noise. a,** Photon-counting imaging results for SBRs of 1/11, 1/56, 1/107, 1/154, 1/245 and 1/332. **b,** The first and the second rows show the QCS images without and with DPIR optimization, respectively.

**Supplementary Note 4. Rotor image reconstruction by the QCS imaging system**

In Fig. 4 of the main text, we present the reconstructed rotor images of a four-rotor drone. To demonstrate the universality of our approach, we also reconstructed images of different drone models (Shantou Xiaowangguo Trading Co., Ltd., 4D-V2), as depicted in Fig. S5. Specifically, Fig. S5(b) shows the photon-counting imaging results when the drone is in the “power off” state, while Fig. S5(c) and (d) show the photon-counting imaging results after pixel expansion through scanning imaging in both the “power off” and “hovering” states. Fig. S5(e) shows a dynamic feature image captured at 211 Hz using the proposed QCS imaging system. Furthermore, Fig. S5(f) displays the spectrum of each pixel along the D (Rotor length) in Fig. S5(e), representing nonzero terms ranging from order 1 to 10. The target time-domain waveform *x* for each pixel is then reconstructed, as shown in Fig. S5(g). Notably, the duty cycle for the time-domain waveform varies from 40.9% at the rotor centre to 6.08% at its edge. Finally, a normalized polar coordinate representation of the drone rotor pattern is presented in Fig. S5(h).


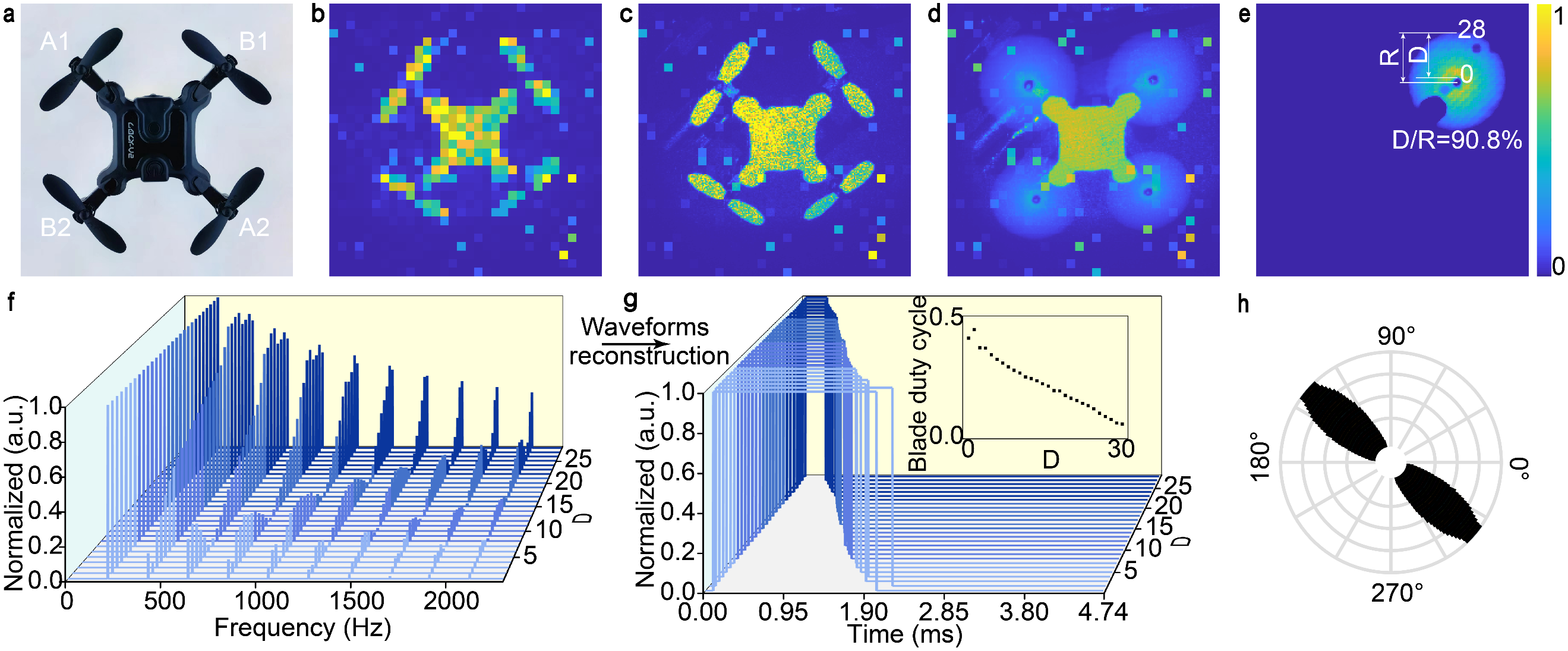


**Fig. S5 Drone rotor image reconstruction by the QCS imaging system. a,** A photograph of the drone. **b,** Photon-counting imaging using the SPAD array. **c, d** Sub-pixel scanning photon-counting images of the drone in the "power off" and "power on" states, respectively. **e,** QCS imaging at 211 Hz. R and D represent the overall diameter and blade length of the drone rotor, respectively. R, Rotor radius. D, Rotor length. **f,** The spectrum of each pixel on rotor length D in (**e**). **g,** The reconstructed time-domain waveform of each spectrum in (**f**). The duty cycle of the blades was calculated using the peaks and troughs of the square wave. **h,** The reconstructed drone rotor pattern.

In order to verify the high coincidence between the reconstructed rotor image and the original image. Fig. S6(a) and (b) show a comparison of the reconstructed rotors shown in Fig. 4(h) in the main text and Fig. S5(h), respectively, with the actual drawing. The results show that they are in good agreement. The width of the reconstructed rotor is slightly wider than that of the real rotor because only a limited number of nonzero terms from the zero-to-tenth-order characteristic frequencies are selected during frequency domain compression sampling. In the time domain, higher-order characteristic frequencies correspond to changes in the rotor details, and omitting higher-order characteristic frequencies leads to an increase in the reconstructed rotor width.

**
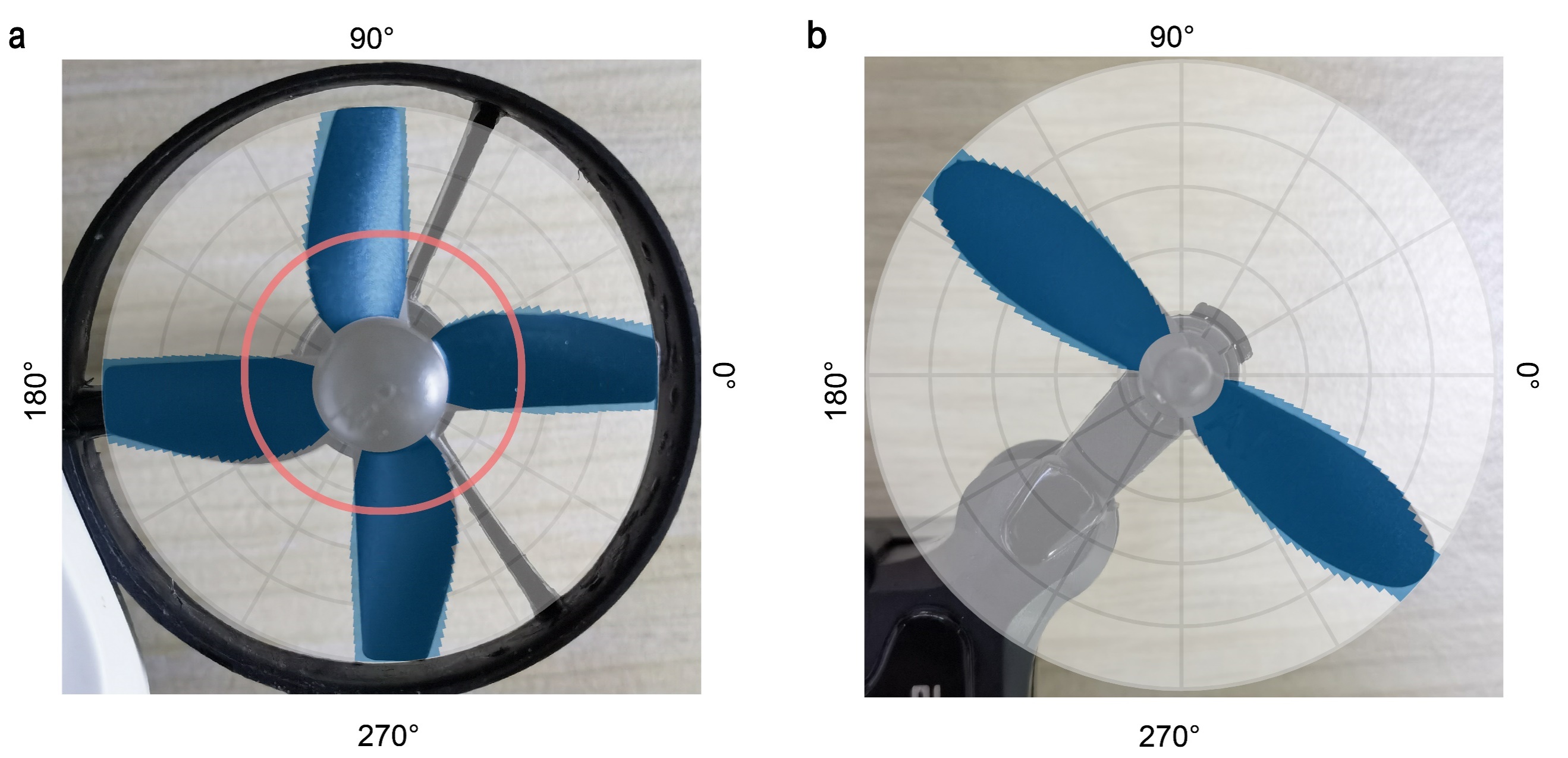
**

**Fig. S6 Comparison of the reconstructed rotor** **patterns and the actual images. a** and **b** show the reconstructed rotor patterns and the actual images in Fig. 4(h) and Fig. S5(h), respectively.

**Supplementary Note 5. The characteristic frequencies of Drones No. 2, No. 3 and No. 4**

Table 1 in the main text shows the characteristic frequencies for the eight flight states of drone No. 1. Here, the characteristic frequencies of the Drones No. 2, No. 3 (Shantou Lingke Culture Communication Co., Ltd., K01) and No. 4 (DJI Marvic 3) are listed in Tables S1, S2 and S3. In this experiment, the characteristic frequencies of 8 fight states of 3 different types of drones were measured respectively. It is verified that the QCS imaging can be used to distinguish different types of drones.

**Table S1: The characteristic frequencies for the eight flight states of Drone No. 2**

| **Drone flight status** | **A1 rotor/Hz** | **A2 rotor/Hz** | **B1 rotor/Hz** | **B2 rotor/Hz** |
| --- | --- | --- | --- | --- |
| Hovering | 260±1 | 263±1 | 262±1 | 262±1 |
| Rise | 979±4 | 1002±10 | 950±4 | 988±5 |
| Turn left | 1112±3 | 1085±3 | 649±4 | 777±3 |
| Turn right | 805±7 | 796±3 | 1020±4 | 1054±2 |
| Move forwards | 944±4 | 902±6 | 938±6 | 858±5 |
| Back | 715±5 | 1009±4 | 954±3 | 966±10 |
| Towards the left | 1008±5 | 926±2 | 869±5 | 1060±4 |
| Towards the right | 827±12 | 1010±4 | 961±6 | 920±5 |

**Table S2: The characteristic frequencies for the eight flight states of Drone No. 3**

| **Drone flight status** | **A1 rotor/Hz** | **A2 rotor/Hz** | **B1 rotor/Hz** | **B2 rotor/Hz** |
| --- | --- | --- | --- | --- |
| Hovering | 263±1 | 277±1 | 282±2 | 272±1 |
| Rise | 993±4 | 978±5 | 838±7 | 935±3 |
| Turn left | 1077±2 | 1099±3 | 660±5 | 745±2 |
| Turn right | 666±2 | 693±3 | 1033±2 | 1013±2 |
| Move forwards | 1013±2 | 868±2 | 953±3 | 826±3 |
| Back | 882±4 | 967±2 | 791±9 | 923±3 |
| Towards the left | 968±5 | 896±3 | 809±7 | 920±1 |
| Towards the right | 875±7 | 1010±4 | 899±6 | 798±4 |

**Table S3: The characteristic frequencies for the eight flight states of Drone No. 4**

| **Drone flight status** | **A1 rotor/Hz** | **A2 rotor/Hz** | **B1 rotor/Hz** | **B2 rotor/Hz** |
| --- | --- | --- | --- | --- |
| Hovering | 178±2 | 152±2 | 188±2 | 148±1 |
| Rise | 221±4 | 161±6 | 215±2 | 151±8 |
| Turn left | 278±2 | 236±10 | 216±6 | 143±5 |
| Turn right | 187±10 | 168±4 | 269±2 | 249±4 |
| Move forwards | 171±5 | 213±2 | 215±7 | 194±4 |
| Back | 228±6 | 259±3 | 145±5 | 171±6 |
| Towards the left | 249±4 | 223±2 | 213±10 | 219±6 |
| Towards the right | 206±4 | 227±3 | 235±5 | 202±8 |

**Supplementary Note 6. QCS imaging of drone**

To more comprehensively evaluate the imaging of the drone with the proposed QCS technology, we obtained images with an upwards view angle. In this experiment, the SPAD array was used in combination with a commercial Schmidt-Cassegrain telescope (AVX925, f=2000 mm, D=200 mm). The Drones No. 4 was vertically launched and hovered at an altitude of 500 m above the experimental system, with the rotor speed set at 5000 rpm. The long focal length of the astronomical telescope resulted in a narrower field of view during imaging, enabling us to discern the general outline of the drone. We captured images of the drone by controlling the imaging device with a high-precision gimbal. Fig. S7 shows the imaging results, where Fig. S7(a) is the photon-counting image of the drone and Fig. S7(b)-(e) show the QCS images of the four rotors. Since the experiment was conducted during the day, there was strong sunlight in the field of view, and the photon-counting imaging system could not distinguish between the target and background noise well. However, the QCS imaging system captured the dynamic characteristics of the target and could determine the characteristic frequency of the drone rotor.


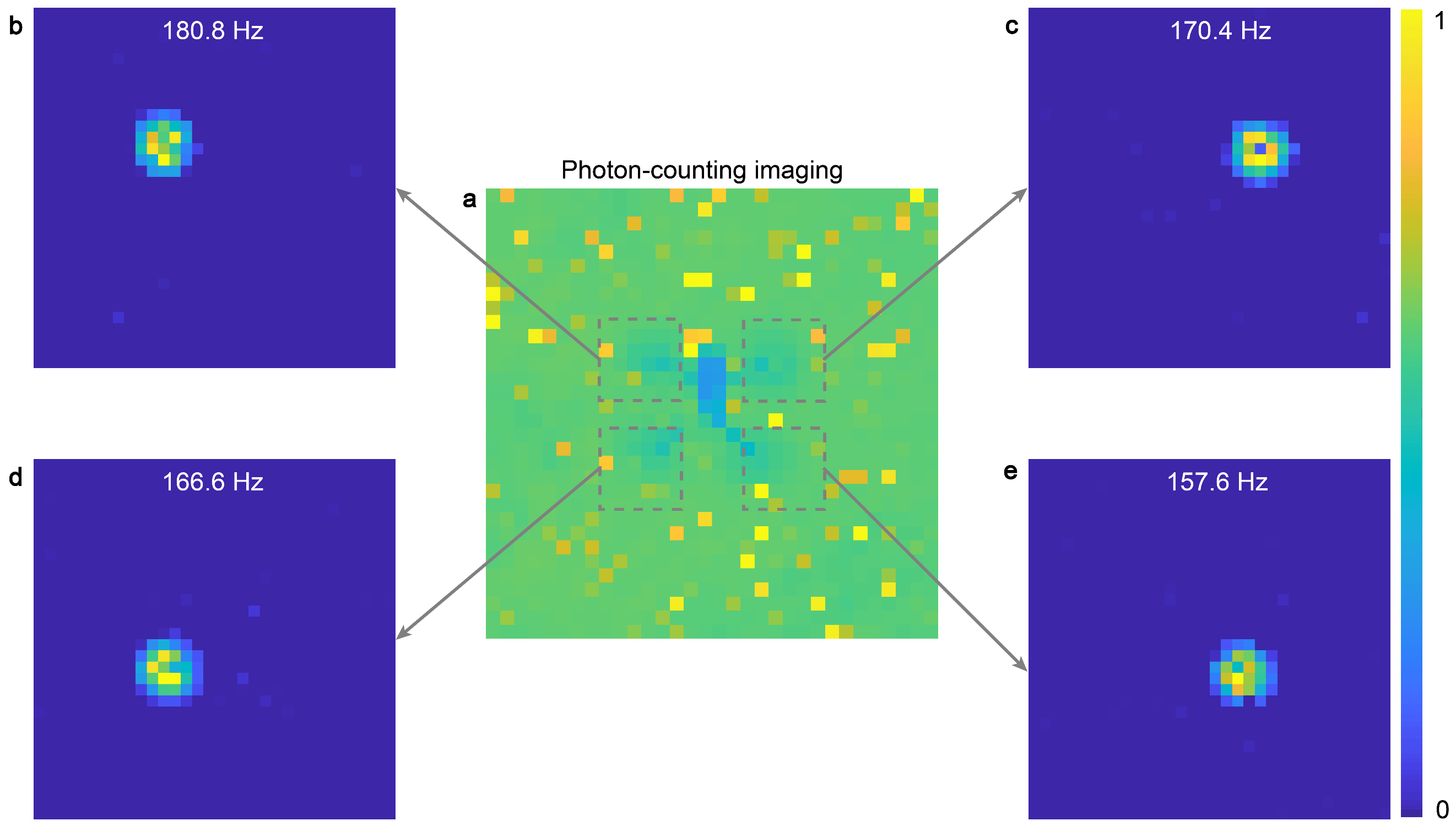


**Fig. S7 Small field-of-view drone imaging at an altitude of 500 m. a,** Photon-counting imaging results of the drone. **b-e,** QCS imaging of the four rotors of the drone and their corresponding characteristic frequency, and their rotor speed can be further calculated.

**Supplementary Note 7. Field test of QCS imaging**

The drone appears as a point target within the imaging field of view when it is flying at a considerable distance. As a point target, the drone cannot be identified directly with traditional passive imaging technology. By employing the QCS imaging, precise point target recognition can be achieved by extracting the dynamic features of the drone at the single-photon level. Another field test was performed in the Twin Pagoda Temple, a famous ancient building in Taiyuan, and the results are presented in Fig. S7. The detector used in the experiment is an SPAD array with a scale of 32×32 pixels. Comprehensive large-scale QCS imaging of the Twin Pagoda Temple was successfully conducted. The captured imagery included trees, residential buildings, and two pagodas, namely, Xuanwen Pagoda and Wenfeng Pagoda. The imaging system can clearly visualize architectural details such as the Sōrin, bracket set, and mullion windows. Concurrently, we positioned a small drone (DJI Mini 3 Pro) adjacent to the top of Wenfeng Pagoda.

The imaging system was positioned 400 m away from the target. We used a digital camera and our experimental setup to acquire images of the selected field-of-view, as shown in Fig. S8(a) and (b) (see Supplementary Note8 for the panoramic photograph taken by the digital camera). Remarkably, the obtained images clearly reproduce the scene. The sōrin, bracket set and mullion window could be clearly distinguished. Fig. S8(c) shows a photograph of Wenfeng Pagoda. Fig. S8(d) and (e) show photon-counting and QCS images of the region where the drone is located, respectively. The overall pixel scale of the image is 8069×2639, and the drone occupies only approximately 2-3 pixels, a very small portion of the field of view. As a result, the drone cannot be identified based on its outline alone. The photon-counting imaging system is susceptible to environmental and system noise, making it challenging to determine the authenticity of the signal. However, the QCS imaging can effectively extract the dynamic characteristics of the target while suppressing background noise. Fig. S8(f) shows the characteristic spectrum in the region where the drone is located, and the observed characteristic frequency of 247 Hz. QCS imaging provides a new strategy for detecting remote drones in strong background environments, enabling model and flight state identification even when the target occupies only a limited number of pixels.


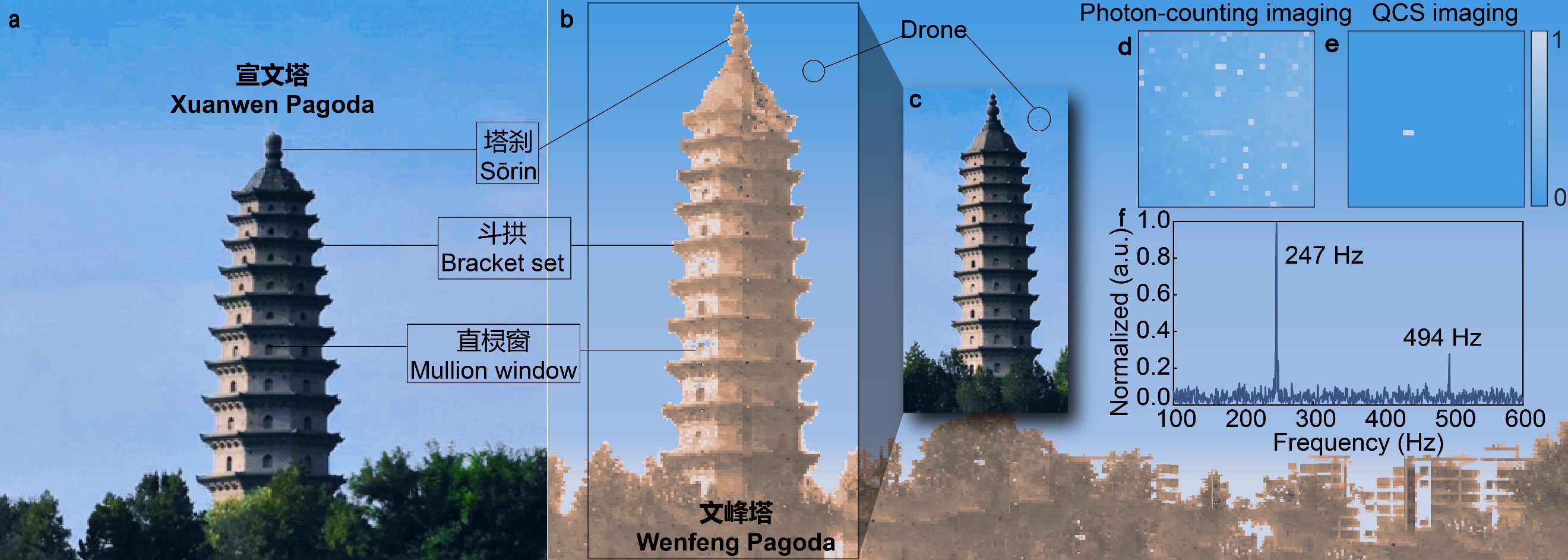


**Fig. S8** **Field test of the QCS imaging system**. **a,** Xuanwen Pagoda photographed by a digital camera (Nikon, D5600). **b,** QCS image of Wenfeng Pagoda. **c,** Wenfeng Pagoda photographed by a digital camera. **d,e,** Photon-counting imaging and QCS imaging result of the drone next to Wenfeng Pagoda. The photon-counting imaging of signal pixels and average background noise are 33 kcps and 34 kcps, respectively. **f,** The characteristic spectrum of the drone rotor.

**Supplementary Note 8. Photograph of the Twin Pagodas Temple**

Fig. S9 in the main text presents a portion of the photograph of the Twin Pagodas Temple. Here, Fig. S8(a) presents a panoramic view of the temple. This photograph was taken on August 31, 2023, at 4:30 pm using a digital camera (Nikon, D5600). The optical system consists of a telescope congregation and Optical elements.

**
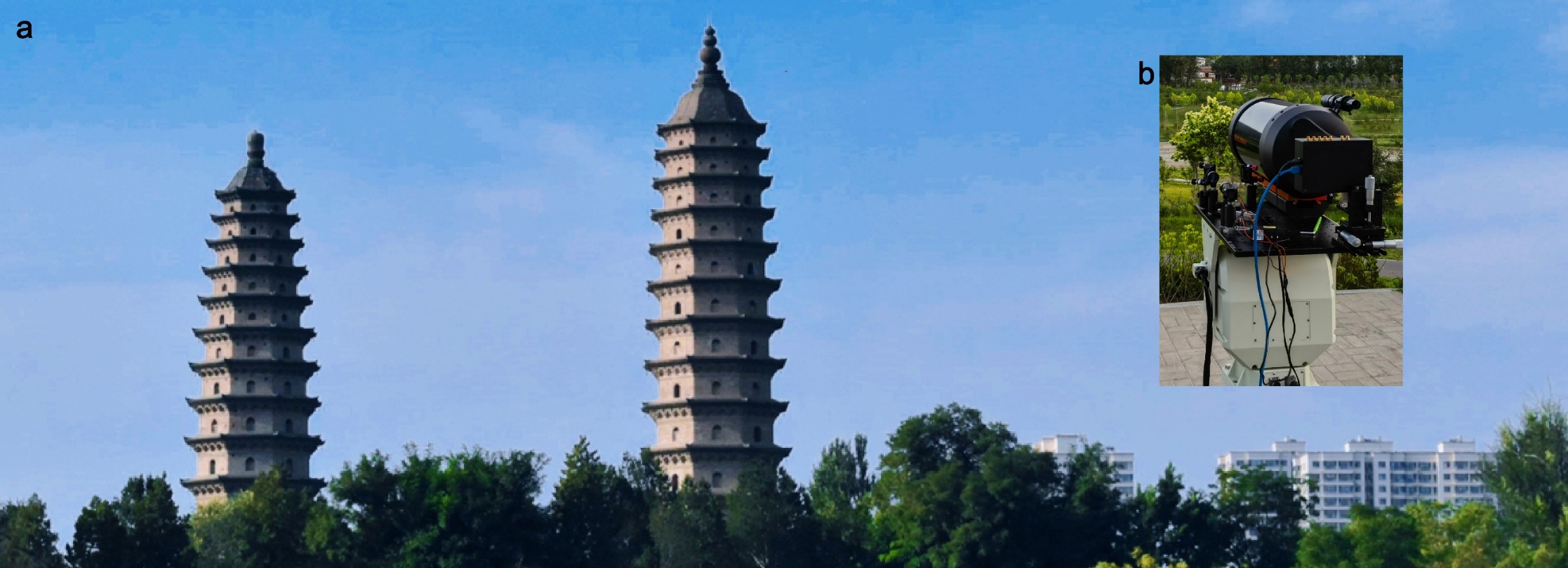
**

**Fig. S9 Photograph of the Twin Pagodas Temple. a,** Photograph of the Twin Pagodas Temple taken with a digital camera. **b,** Photograph of the setup. The optical system is fixed to the heavy duty PTZ.

**Supplementary Note 9. Different perspectives for observing drone rotors**

The perspectives from which drones are observed can be categorized into three primary types, as illustrated in Fig. S10:

(1) The modulation of the illumination light by the rotation of the drone's rotor is easy to understand from the top view and the upward view. In these views, the rotor presents its largest reflective cross-section to the light, resulting in maximum modulation depth, as illustrated in Fig. S10(b) and (c). Consequently, under identical lighting conditions, this perspective maximizes the detection signal-to-noise ratio (SNR).

(2) When the observation perspective is a side view, the illumination light is still modulated by the rotation of the drone's rotor. As shown in Fig. S10(d), which depicts three typical rotor positions during side-view observation, these states alternate continuously, but the modulation depth of the light is reduced due to the relatively small reflection cross-section. Consequently, under identical lighting conditions, the detection SNR decreases in this observation perspective. Of course, if the rotor is guarded, it is impossible to observe the dynamic frequency information.

(3) In actual experiments, the observation perspective often lies between a upward view and a side view, as indicated by the red arrow in Fig. S10(a). From this perspective, the reflective cross-section of the rotor lies in between. Consequently, under identical lighting conditions, the detection SNR also falls within an intermediate range.

In this work, the observation point is located 10 km from the drone's take-off position. The elevations of the observation and take-off positions are 800 m and 1214 m, respectively, as measured by a GPS surveyor, resulting in an elevation difference of 414 m. Considering the hovering altitude of the drone, the calculated view angle is 2.4°. Despite the reduced detection SNR compared to top or upward views, it remains possible to capture the dynamic characteristics of the drone's rotor.

**
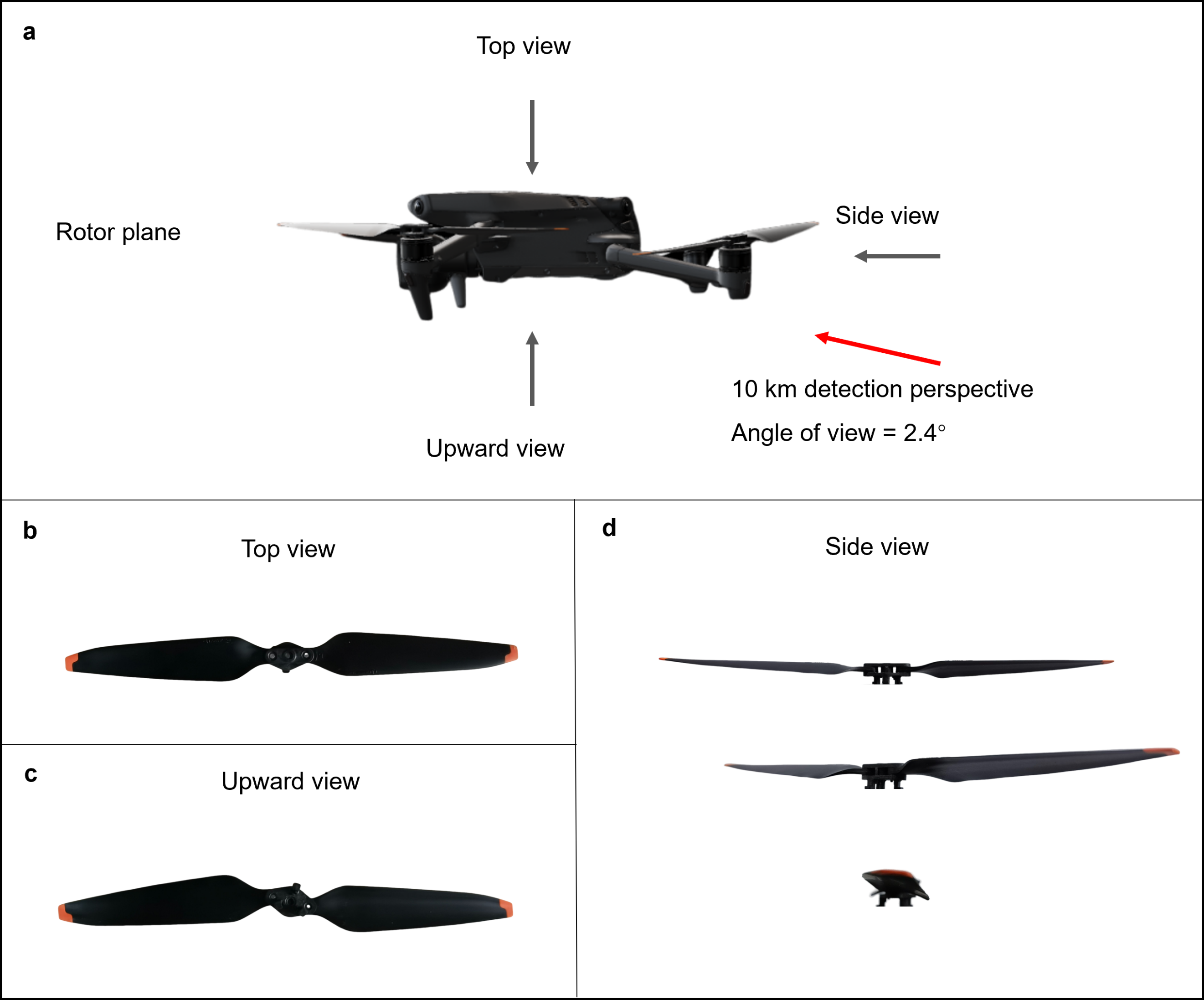
**

**Fig. S10** **Different perspectives for observing drone rotors. a** Three typical observation drone perspectives and a 10 km detection perspective. **b-d** Schematic diagrams of the rotor in top, upward, and side views. The three states are alternately cycled in the side view.

**References:**

1. Donoho DL. Compressed sensing. *IEEE Trans. Inf. Theory* **52,** 1289-1306 (2006).

2. Rani M, Dhok SB, Deshmukh RB. A Systematic Review of Compressive Sensing: Concepts, Implementations and Applications. *Ieee Access* **6,** 4875-4894 (2018).

3. Hu JY, Yu B, Jing MY, Xiao LT, Jia ST, Qin GQ*, et al.* Experimental quantum secure direct communication with single photons. *Light-Sci. Appl.* **5,** (2016).

4. Hu JY, Jing MY, Zhang GF, Qin CB, Xiao LT, Jia ST. Performance of single-photons communication using the multi-channel frequency coding scheme. *Opt. Express* **26,** 20835-20847 (2018).

5. Hu JY, Liu Y, Liu LL, Yu B, Zhang GF, Xiao LT*, et al.* Quantum description and measurement for single photon modulation. *Photonics Res.* **3,** 24-27 (2015).

6. Zhang K, Li YW, Zuo WM, Zhang L, Van Gool L, Timofte R. Plug-and-Play Image Restoration With Deep Denoiser Prior. *IEEE Trans. Pattern Anal. Mach. Intell.* **44,** 6360-6376 (2022).
